# Runx1 and Runx3 Are Involved in the Generation and Function of Highly Suppressive IL-17-Producing T Regulatory Cells

**DOI:** 10.1371/journal.pone.0045115

**Published:** 2012-09-12

**Authors:** Lequn Li, Nikolaos Patsoukis, Victoria Petkova, Vassiliki A. Boussiotis

**Affiliations:** 1 Department of Medicine, Division of Hematology-Oncology and Cancer Biology, Beth Israel Deaconess Medical Center, Harvard Medical School, Boston, Massachusetts, United States of America; 2 Department of Pathology, Beth Israel Deaconess Medical Center, Harvard Medical School, Boston, Massachusetts, United States of America; McGill University Health Center, Canada

## Abstract

CD4^+^Foxp3^+^ T regulatory cells (Tregs) display phenotypic and functional plasticity that is regulated by cytokines and other immune cells. Previously, we determined that during co-culture with CD4^+^CD25^−^ T cells and antigen presenting cells, Tregs produced IL-17. Here, we investigated the mechanisms underlying the differentiation of IL-17-producing Treg (Tr17) cells and their molecular and functional properties. We determined that during stimulation via TCR/CD3 and CD28, the combination of IL-1β and IL-2 was necessary and sufficient for the generation of Tr17 cells. Tr17 cells expressed Runx1 transcription factor, which was required for sustained expression of Foxp3 and RORγt and for production of IL-17. Surprisingly, Tr17 cells also expressed Runx3, which regulated transcription of perforin and granzyme B thereby mediating cytotoxic activity. Our studies indicate that Tr17 cells concomitantly express Foxp3, RORγt, Runx1 and Runx3 and are capable of producing IL-17 while mediating potent suppressive and cytotoxic function.

## Introduction

Tight regulation of effector T cell responses is required for effective control of infections and avoidance of autoimmunity. Tregs have essential roles in the maintenance of immune homeostasis. During the past few years a series of studies have shown that subsets of Tregs have the propensity to differentiate into Th17 or Tfh cells [1], [2], [3], [4]. By genetic fate mapping using Foxp3-Cre mice to mark cells that expressed Foxp3 at some stage of development, at least one quarter of small intestinal IL-17 producing cells were found to express Foxp3 during their ontogeny [5]. It has been proposed that this might be the outcome of stochastic activation of Foxp3 expression or, alternatively, the co-expression of Foxp3 and IL-17 might mark a distinct differentiation pathway [6]. IL-17^+^Foxp3^+^ T cells not only have been observed under experimental conditions but have been identified in the inflamed intestinal mucosa of patients with Crohn's disease and are associated colon cancer in the context of ulcerative colitis [7], [8]. These findings strongly suggest that differentiation of IL-17^+^ Tregs is a natural process that can occur under physiologic or immunopathologic conditions in vivo.

Foxp3 plays a negative role in IL-17 expression through physical interaction with Runx1 and RORγt, thereby inhibiting their transactivation activity [5], [9]. Runx1 is highly expressed in CD4^+^ T lymphocytes [10] as well as Foxp3^+^ Tregs and interaction between Runx1 and Foxp3 is essential for the function of Foxp3 [11]. Runx1 also has a significant role in Th17 differentiation due to its ability to induce expression of RORγt and to associate with and to act together with RORγt to induce *Il17* transcription [12]. Runx3 also regulates expression of Foxp3 [13], [14]. However, in contrast to Runx1 that is expressed on CD4^+^ T cells, Runx3 is predominantly expressed in CD8^+^ T cells, and cooperates with T-box proteins to establish the transcriptional program of effector cytotoxic T lymphocytes [15].

We have previously determined that during co-culture with CD4^+^CD25^−^ T cells in the presence of antigen presenting cells (APC), a fraction of Tregs differentiated into IL-17-producing, Tr17 cells [16]. In the present study we examined the mechanisms underlying the differentiation of Tr17 cells and their molecular and functional properties.

## Results

### Tr17 cells retain phenotypic properties of Tregs

**Figure 1:**
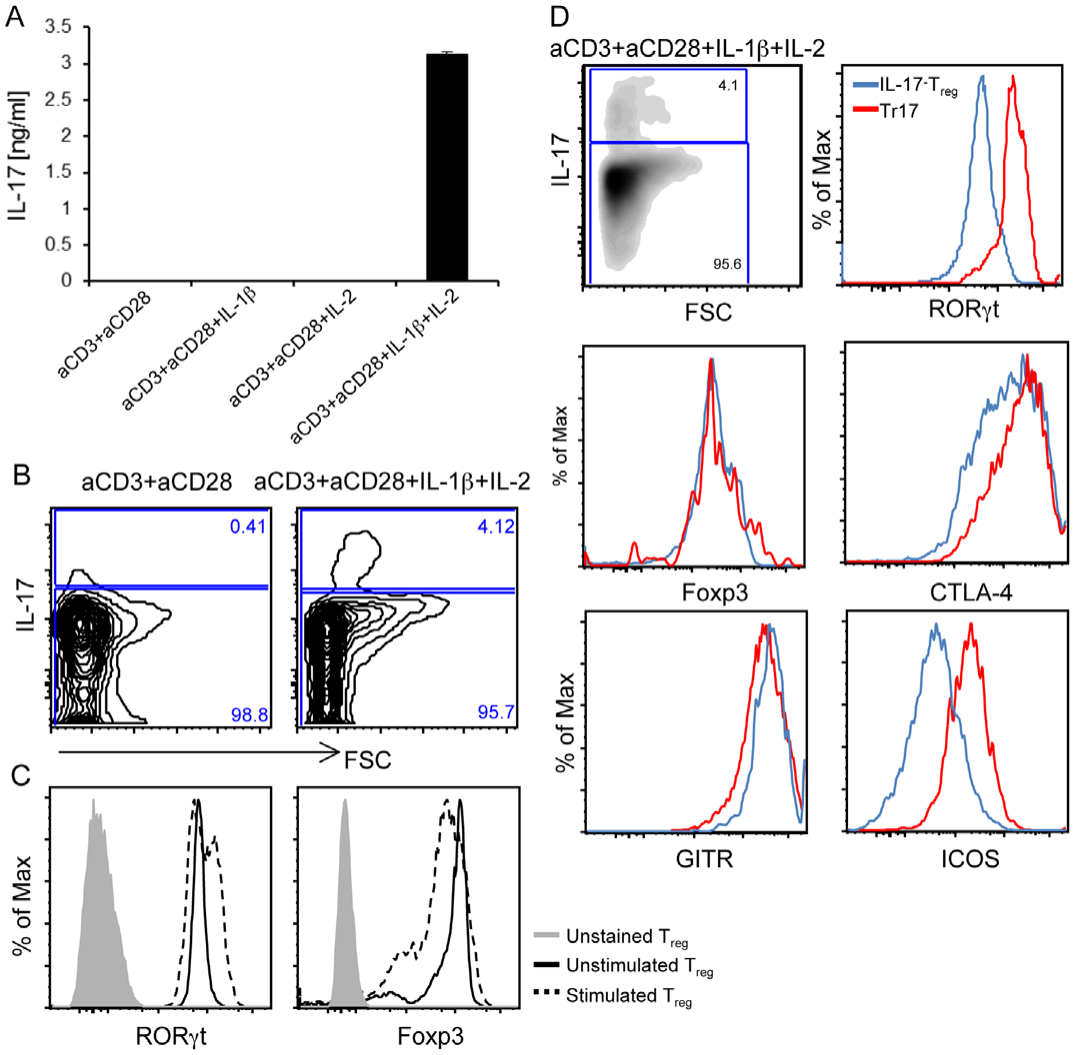
Generation and immunophenotype of Tr17 cells. (*A*) Tregs isolated from Foxp3.GFP knock in mice were stimulated with anti-CD3/CD28 mAbs alone or in the presence of IL-1β, IL-2 or their combination. Production of IL-17 was measured by ELISA (eBioscience). Data was representative of five independent experiments. (*B*) Tregs were cultured with anti-CD3/CD28 alone or in the presence of IL-1β and IL-2 (polarizing conditions), and were subsequently stimulated with PMA and ionomycin for additional 4 h, followed by intracellular staining with anti-IL-17 antibody. Numbers indicate percent IL-17^+^ cells (upper box) and IL-17^−^ cells (lower box) within total live cells. (*C*) Expression of RORγt and Foxp3 in freshly isolated Tregs (Unstimulated) and in Tregs cultured under polarizing conditions (Stimulated) was assessed by intracellular staining. (*D*) Expression of the indicated transcription factors and surface markers was analyzed on gated IL-17^+^ and IL-17^−^ Tregs. The data (*B–D*) were representative of three independent experiments.

We have previously reported that during co-culture of CD4^+^CD25^−^ cells and Tregs in the presence of APC, IL-1β and IL-2, endogenous products of the co-culture, were essential for the generation of Tr17 cells [16]. To determine whether these cytokines were necessary and sufficient to induce differentiation of Tr17 cells, we used Foxp3.GFP knock-in mice to isolate highly purified Tregs. CD4^+^Foxp3^+^ Tregs were cultured in an APC-free system using anti-CD3/CD28 mAbs in the presence of IL-1β, IL-2 or their combination. A significant amount of IL-17 secretion was observed only when a combination of IL-1β and IL-2 was added (thereafter termed polarizing culture conditions) (Fig. 1*A*). Intracellular staining confirmed the generation of IL-17 producing Tregs (Fig. 1*B*). Because IL-17 secretion is associated with expression of RORγt [17], we assessed the expression of RORγt. Activation of Tregs under polarizing conditions resulted in upregulation of RORγt, while expression of Foxp3 was retained (Fig. 1*C*). Tr17 cells expressed significantly higher levels of RORγt compared to Tregs that remained IL-17^−^ during polarizing culture (Fig. 1*D* and Fig. S1), consistent with previous reports on IL-17^+^Foxp3^+^ T cells identified in different systems [18], [19]. Thus although the expression of Foxp3 remained unchanged, the level of RORγt expression in Tregs positively correlated with their ability to produce IL-17.

To determine whether Tr17 cells retained phenotypic properties of Tregs we examined well-established markers. Expression of CTLA-4 and GITR was comparable between Tr17 and IL-17^−^ Tregs (Fig. 1*D*). We also examined expression of ICOS that has been suggested to define distinct subsets of Tregs [20]. Interestingly, Tr17 cells displayed increased ICOS levels as compared to IL-17^−^ Tregs (Fig. 1*D*), suggesting that ICOS expression might identify Tregs that are capable of producing IL-17. Tr17 cells also expressed Helios (Fig. S2), a marker previously proposed to identify subsets of Treg cells [21], [22].

### Expression of Runx1 is required for the differentiation of Tr17 cells

**Figure 2:**
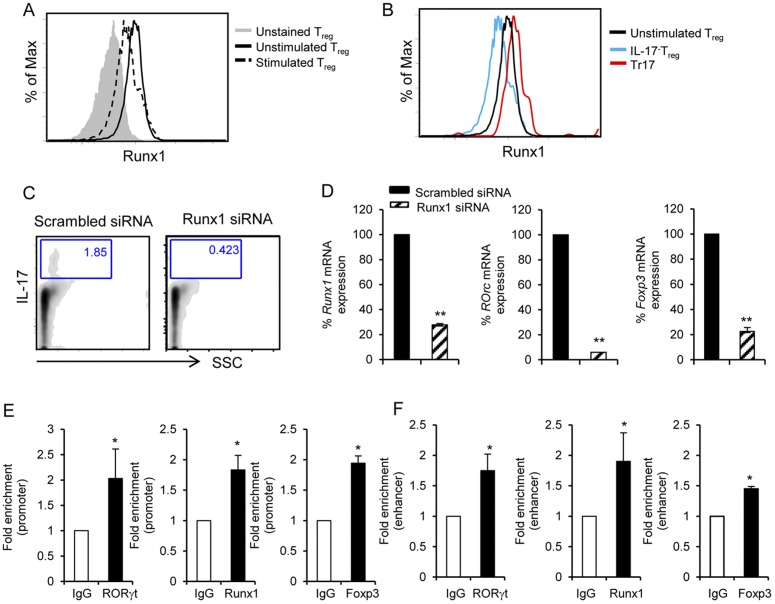
Expression of Runx1 is required for the generation of Tr17 cells. (*A*) Expression of Runx1 in freshly isolated Tregs (Unstimulated) and in Tregs cultured under polarizing conditions (Stimulated) was assessed by intracellular staining. (*B*) After four days of polarizing culture, expression of Runx1 was analyzed on gated IL-17^+^ and IL-17^−^ Tregs. (*C*) Tregs were transfected with Runx1-specific siRNA or control (scrambled) siRNA and were cultured under polarizing conditions followed by intracellular staining for IL-17. The data are representative of two independent experiments. (*D*) In the same cells as in (C), expression of *Runx1*, *Rorc* and *Foxp3* was examined by real time PCR. Bar graphs show results from one out of two independent experiments. (*E, F*) Binding of RORγt, Runx1 and Foxp3 to the *Il17* promoter (*E*) and enhancer (*F*) was assessed by ChIP with antibodies against RORγt, Runx1 and Foxp3 in Tregs cultured under polarizing conditions and restimulated for 4 h with PMA and ionomycin. Results were normalized to input and are shown as fold increase in signal relative to isotype control IgG used as ChIP negative control. Results from one out of two experiments are shown.

Our results showed that Tr17 cells expressed enhanced levels of RORγt while retaining expression of Foxp3. To understand the mechanism underlying this unique property of Tr17 cells, we focused on Runx1, a transcription factor that can regulate the expression of both *Rorc* and *Foxp3*. Expression of Runx1 in bulk Tregs was downregulated after culture with anti-CD3/CD28 mAbs (Fig. S3), similarly to previous observations in naïve CD4^+^ T cells [10]. Expression of Runx1 was also downregulated in bulk Treg polarizing cultures but to a lower extend (Fig. 2*A* and Sig. S3). Strikingly, after gating on IL-17 producer Tregs we determined that Tr17 cells did not display Runx1 downregulation but, instead, had increased Runx1 expression compared to untreated Tregs. In contrast, Runx1 was downregulated in IL-17^−^ Tregs (Fig. 2*B*). These results indicate that the increased Runx1 expression in Tr17 cells accounted for the less prominent downregulation of Runx1 expression in bulk Treg polarizing cultures. To determine the role of Runx1 in the generation of Tr17 cells, we attenuated expression of Runx1. Silencing of Runx1 with small interfering RNA (siRNA) during polarizing culture, significantly reduced the numbers of IL-17^+^ cells (0.423%) compared to transfection with control ‘scrambled’ siRNA (1.85%) (Fig. 2*C*), suggesting that Runx1 plays an important role in regulating IL-17 production in Tregs. Moreover, expression of *Rorc* and *Foxp3* mRNA was significantly reduced in cells transfected with Runx1-specific siRNA (Fig. 2*D*). Thus, Runx1 plays an essential role in the generation of Tr17 cells by regulating expression of both *Rorc* and *Foxp3* transcription factors.

Previous studies in Th17 cells have identified the IL-17 promoter and one conserved noncoding (enhancer) sequence, CNS-5, as the regions required for the optimal transcription of the *Il17* gene [12]. Both these regulatory elements contain sequences that bind the transcription factors RORγt and Runx1. Because Tr17 cells express Foxp3, which has the capacity to interact with RORγt and Runx1, we examined whether this physical interaction might interfere with the ability of these IL-17 regulatory transcription factors to bind on the relevant regulatory elements of the *Il17* gene, using chromatin immunoprecipitation (ChIP). Binding of RORγt and Runx1 to the *Il17* promoter and enhancer was detected in Tregs cultured under polarizing conditions (Fig. 2*E, F*). However, binding of RORγt and Runx1 to these elements in Tr17 was diminished compared to Th17 cells (Fig. S4). To determine whether Foxp3 might indeed form complexes with RORγt and/or Runx1 bound on the *Il17* promoter and enhancer, we did ChIP using Foxp3-specific antibody. Foxp3 displayed binding to the *Il17* promoter and enhancer in Tr17 cells (Fig. 2*E and F*) but not in Th17 cells (Fig. S4). Because no Foxp3 binding sites are present on the *Il17* promoter and enhancer [12], these findings suggest that Foxp3 was detected on these elements due to complex formation with RORγt and/or Runx1 bound on these regions of the *Il17* gene.

### Tr17 cells display potent suppressive activity

**Figure 3:**
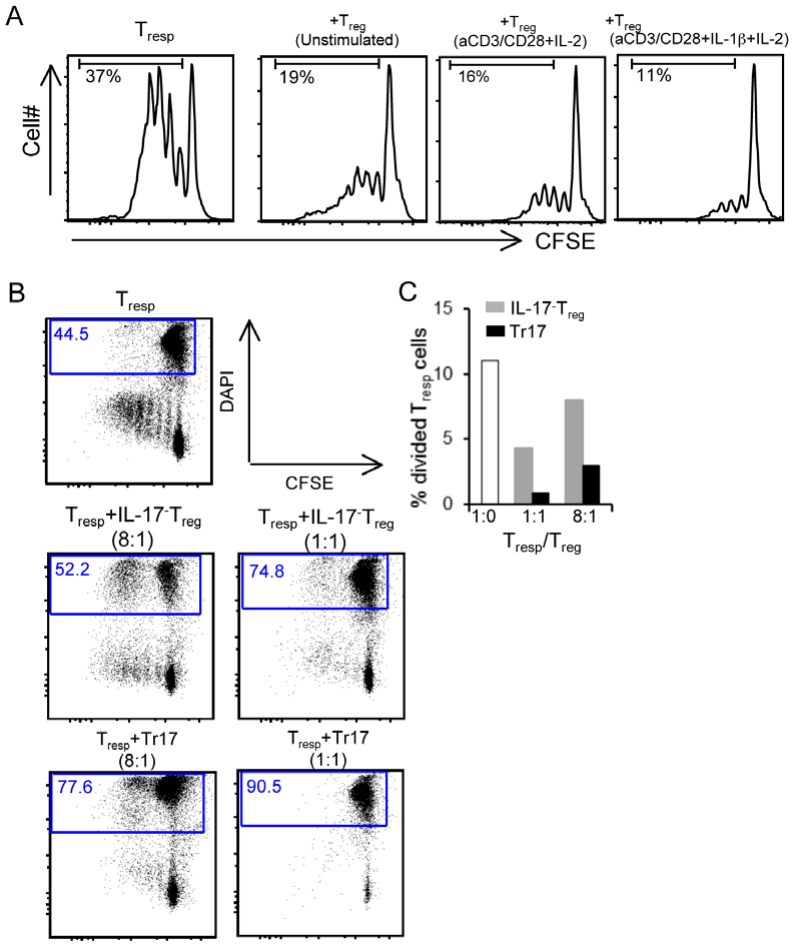
Tr17 cells display potent suppressive activity. (*A*) CFSE-labeled CD4^+^CD25^−^ T cells (Tresp) from PL mice expressing Thy1.1 were cultured with anti-CD3/CD28 either alone or with indicated Tregs and cell divisions were assessed by CFSE dye dilution. The numbers indicate percent proliferating cells within total live Thy1.1^+^ cells (Tresp). (*B*) Tregs were cultured under polarizing conditions and IL-17^+^ and IL-17^−^ cells were sorted by cytokine capture and flow cytometry. CFSE-labeled Tresp cells from PL mice were cultured with anti-CD3/CD28 either alone or with Tr17 or IL-17^−^ Tregs for 4 d. Cell proliferation and death of Thy1.1^+^ Tresp cells were simultaneously assessed by co-staining with DAPI. Numbers indicate percent of dead cells within the Thy1.1^+^ population. (*C*) Proliferation of DAPI^−^ Tresp cells was analyzed by flow cytometry. The data (*A–C*) are representative of three independent experiments.

To determine whether Tr17 retained their functional role as Tregs, we performed suppression assay. Upon culture of CD4^+^CD25^−^ responder T cells (Tresp) with anti-CD3/CD28 mAbs, approximately 37% of Tresp underwent at least one round of cell division (Fig. 3*A*, first panel). Only 19% of Tresp divided when they were co-cultured with freshly isolated Tregs (Fig. 3*A*, second panel) and 16% of Tregs divided when they were co-cultured with Treg that were previously stimulated with anti-CD3-plus-anti-CD28 *in vitro* (Fig. 3*A*, third panel). Interestingly, further reduced proportion of Tresp (11%) proliferated when co-cultured with Treg that had been previously incubated under polarizing culture conditions (Fig. 3*A*, forth panel), suggesting that the inhibitory capacity of these Tregs was enhanced. In order to analyze more accurately the suppressive function of Tr17 cells, we used cytokine capture to isolate highly purified populations of IL-17+ (Tr17) and IL-17− Tregs. Suppression assay indicated that Tr17 cells displayed more potent inhibitory function compared to IL-17− Tregs and this effect was dose dependent (Fig. 3*B, C*). Specifically, at Treg: Tresp ratio of 1∶8, co-culture of Tr17 with Tresp led to only 3% of Tresp cells undergoing cell division, whereas twice as many Tresp proliferated when co-cultured with IL-17^−^ Tregs. Furthermore, at Treg: Tresp ratio of 1∶1, co-culture of Tr17 with Tresp led to almost undetectable response. During this analysis, we noted an increased percentage of dead cells in the co-cultures of Tresp with Tr17 as compared to IL-17− Tregs. To distinguish between anti-proliferative and cytotoxic effects of Tregs, we simultaneously assessed induction of cell death in CFSE-labeled Tresp co-stained with DAPI. There was a dose-dependent increase in Tresp cell death when co-cultured with Tregs and this effect was more prominent upon co-culture with Tr17 cells (Fig. 3*B*).

### Perforin and granzyme B are highly expressed in Tr17 cells

**Figure 4:**
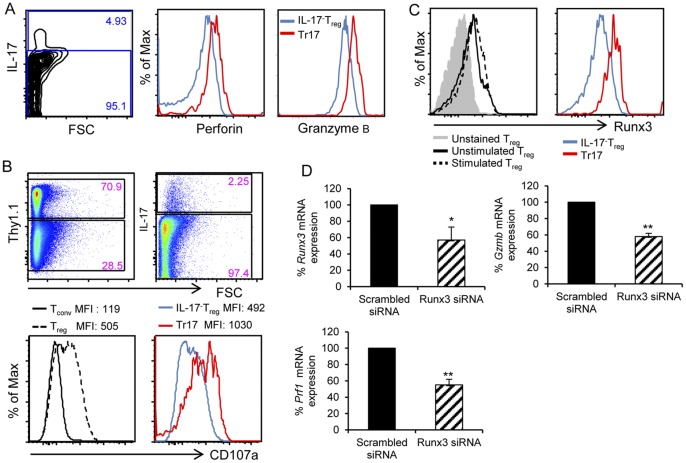
Expression of perforin, granzyme B and Runx3 in Tr17 cells. (*A*) Tregs were cultured under polarizing conditions and were restimulated with PMA and ionomycin for 4 h, followed by intracellular staining with antibodies specific for IL-17, perforin and granzyme B and expression was analyzed in IL-17^+^ and IL-17^−^ cells. Results are representative of 4 independent experiments. (*B*) Tregs cultured under polarizing conditions were harvested and co-cultured with CD4^+^CD25^−^ Thy1.1^+^ Tresp cells and anti-CD3/CD28 in the presence of PE-conjugated antibody against CD107a for 2 h. The left histograms (upper and lower) show expression of CD107a on Tresp and Treg cells; the right histograms (upper and lower) show expression of CD107a in IL-17^−^ Treg and Tr17 cells. (*C*) After culture under polarizing conditions, expression of Runx3 in bulk Treg (left panel) and in gated IL-17^+^ or IL-17^−^ Treg (right panel) was analyzed by intracellular staining. (*D*) Tregs transfected with scrambled siRNA or Runx3-specific siRNA were cultured under polarizing conditions for 48 h and expression of *Runx3*, *Prf1* and *Gzmb* mRNA was examined by real-time PCR. Results from one out of two experiments are shown.

One of the proposed mechanisms for Treg-mediated suppression involves cytolysis of Tresp cells via the granzyme B-perforin pathway [23], [24], [25]. We investigated whether Tr17 cells expressed granzyme B and perforin and utilized this pathway to induce cytolysis of Tresp. Flow cytometry analysis revealed that Tr17 cells expressed higher levels of perforin and granzyme B compared to IL-17^−^ Tregs (Fig. 4*A*). To identify the degranulation capacity of Tr17 cells we assessed CD107a (LAMP-1), a marker of degranulation in CD8^+^ and NK cells [26], [27]. After polarizing culture, Tregs were co-cultured with Tresp isolated from B6 congenic mice (Thy1.1^+^) and expression of CD107a was examined. Mean fluorescence intensity (MFI) of CD107a in Tregs was 4-fold higher than in Tresp cells (Fig. 4*B*, bottom, left panel). Furthermore, expression of CD107a in Tr17 cells was significantly enhanced compared to IL-17^−^ Tregs (Fig. 4*B* bottom, right panel), indicating that degranulation is a function preferentially exerted by Tr17 cells.

Runx3 plays a critical role in CTL differentiation and regulates expression of *Gzmb* and *Prf1* either as a sole factor or in synergy with other transcription factors [15]. Expression of Runx3 was low in freshly isolated Tregs and was only slightly upregulated in bulk Tregs during polarizing culture (Fig. 4*C*). However, assessment of Runx3 on Tr17 and IL-17^−^ Tregs indicated that Tr17 cells expressed significantly higher levels of Runx3 protein (Fig. 4*C*). To determine whether Runx3 had a causative role in regulating expression of granzyme B and perforin in Tregs, we used Runx3-specific siRNA. Runx3 siRNA reduced Runx3 mRNA expression during polarizing culture by 47% (Fig. 4*D*). A comparable reduction was also observed in granzyme B and perforin mRNA (Fig. 4*D*) indicating that Runx3 had an active role in regulating these genes in Tr17 cells.

## Discussion

In our present study we determined that similarly to Th17 cells, in Tr17 cells Runx1 and RORγt bind on the previously identified binding elements of the *Il17* gene but the abundance of these transcription factors on the relevant DNA elements is significantly lower. Tr17 cells express Foxp3, which plays a negative role in IL-17 expression because via its physical interaction with RORγt and Runx1 inhibits their transactivation activity [5], [12]. Our findings strongly suggest that the reduced abundance of RORγt and Runx1 on the IL-17 promoter and enhancer of Tr17 cells might be related to their association with Foxp3, which was also detected in the same DNA elements by CHIP in Tr17 but not in Th17 cells.

Runx1 and Runx3 transcription factors play key roles in T cell development and differentiation [28]. Runx1 is highly expressed in resting CD4^+^ T cells but signals mediated by TCR during activation rapidly downregulate Runx1 via negative autoregulation of the distal *Runx1* promoter [10]. Runx1 plays an essential role in the generation and function of Treg and Th17 cells [11], [12]. It was previously shown that Runx1 regulates *Il17* transcription in CD4^+^ T cells [12]. However, in that study the expression pattern of endogenous Runx1 during differentiation of CD4^+^ T cells to Th17 was not investigated. In our studies we examined the expression pattern of endogenous Runx1 in Tregs during their differentiation to Tr17. We found that in the presence of IL-1β and IL-2, expression of Runx1 not only was retained but was even increased compared to unstimulated Tregs. Furthermore, silencing Runx1 expression by siRNA reduced the number of Tr17 cells. Our results strongly suggest that sustained expression of Runx1 in Tregs is regulated by polarizing cytokines and is required for the differentiation of Tregs to Tr17 cells.

An additional novel and unexpected finding of our studies was that Tr17 cells expressed increased levels of perforin and granzyme B, and displayed enhanced cytotoxic activity compared to IL-17^−^ Tregs. Furthermore, Tr17 cells displayed increased expression of Runx3, which is involved in the expression of *Prf1* and *Gzmb* genes, thereby regulating cytolytic function. Unlike Runx1, Runx3 is not expressed in naïve CD4^+^ T cells but is induced during Th1 differentiation [29]. Runx3 is also expressed in CD8^+^ T cells and regulates transcription of *Prf1* and *Gzmb* through binding on multiple regulatory regions of both genes, including their transcription start sites [15]. Runx3 has no effect on the differentiation of Th17 cells [12]. In contrast to this finding in Th17 cells, our results showed increased expression of Runx3 in Tr17 cells, which was functionally important. In CD8^+^ T cells, expression of Runx3 requires the presence of T-bet and is necessary for the induction of Eomes. In that setting, Runx3 cooperates with T-bet to regulate transcription of granzyme B and with Eomes to regulate transcription of perforin [15]. Further studies will determine whether Runx3 is downstream of T-bet and whether it regulates expression of Eomes in Tr17 cells.

The functional efficacy of Tregs in vivo is programmed by environmental cues. Such functional adaptation may improve the ability of Tregs to control different classes of immune responses driven by distinct types of T effector cells. For example, in response to interferon-γ Tregs express increased levels of Th1-specific transcription factor T-bet, leading to expression of the chemokine receptor CXCR3, which enables T-bet^+^ Tregs to share similar migratory properties with Th1 cells and to accumulate at the same sites thereby controlling Th1-mediated inflammation [30]. It is intriguing to speculate that Tr17 cells might be differentiated in an inflammatory microenvironment and might be programmed to regulate Th17-mediated inflammatory responses. In conclusion, our studies indicate that Tr17 cells concomitantly express Foxp3, RORγt, Runx1 and Runx3 and are capable of producing IL-17 while mediating potent suppressive function. These findings might have significant implications in our understanding of how environmental cues translate into determinants of Treg fate and might guide the development of therapeutic strategies that use the administration of Tregs for the treatment of autoimmune diseases.

## Materials and Methods

### Ethics Statement

All experiments were conducted in accordance with National Institutes of Health guidelines. The studies were reviewed and approved by the Institutional Animal Care and Use Committee (Approved Protocol Number: #122-2010).

### Mice

Foxp3-GFP knock-in (C57BL/6 background) from Dr. Mohamed Oukka (Brigham and Women's Hospital, Boston, MA), C57/B6 (Charles River, MA) and B6.PL (The Jackson Laboratory, Bar Harbor, ME) were used.

### Cell culture

CD4^+^ T cells were isolated using CD4^+^ T Cell Isolation Kit (Miltenyi Biotec) and CD4^+^Foxp3.GFP^+^ Tregs were sorted by flow cytometry. Tregs (1×10^6^ cells/ml) were stimulated with anti-CD3 (1 μg/ml) and anti-CD28 (1 μg/ml) mAbs alone or in the presence of IL-1β (10 ng/ml), IL-2 (10 ng/ml) or both for 4 days. For differentiation of Th17 cells, CD4^+^ T cells (1×10^6^ cells/ml) were cultured with anti-CD3/anti-CD28 mAbs in the presence of TGF-β (2 ng/ml) and IL-6 (20 ng/ml).

### Suppression Assay

To assess suppressive activity of IL-17^−^ Tregs and Tr17 cells, after polarizing culture IL-17^+^ cells were enriched by cytokine capture using Mouse IL-17 Secretion Assay kit (Miltenyi Biotec) according to the manufacturer's protocol. Flow cytometry-sorted IL-17^+^GFP^+^ and IL-17^−^GFP^+^ cells were used for suppression assay using as responder CD4^+^CD25^−^ cells from B6.PL congenic mice labeled with CFSE and DAPI. Percent of dividing cells was calculated using a described formula [31].

### Degranulation Assay

Tregs were stimulated with PMA plus ionomycin for 4 h and were cultured with CD4^+^ T cells from B6.PL mice and anti-CD3/CD28 mAbs in the presence of PE-conjugated antibody against CD107a (BD Pharmingen, clone 1D4B). Cells were collected, stained with antibodies against Thy1.1 and IL-17 and analyzed by flow cytometry.

### RNA-mediated interference

For knockdown of Runx1 and Runx3, CD4^+^Foxp3.GFP^+^ cells were transfected by nucleofection (Amaxa; X-01 Program) with siRNA specific for Runx1, Runx3 or ‘scrambled’ control siRNA (Dharmacon). After 48 h of culture, RNA was extracted and analyzed by real-time RT-PCR for expression of *Runx1*, *Rorc*, *Foxp3*, *Runx3*, *Prf1* and *Gzmb*. Real time PCR primers and probes were obtained from Applied Biosystems.

### ChIP assay

Tregs and CD4^+^ T cells were cultured for 4 days under the indicated conditions and ChIP assay was performed using Pierce Agarose ChIP kit (Thermo Scientific) according to manufacturer's instructions. Antibodies used for IP were specific for Runx1 (Active Motif), RORγt (Santa Cruz Biotechnology), Foxp3 (Santa Cruz Biotechnology) or IgG isotype control.

### Statistical analysis

To assess statistical significance of quantitative differences of various parameters, t-test was used. Differences were considered statistically significant for p values <0.05 (* corresponds to p<0.05; ** corresponds to p<0.001).

## Supporting Information

Figure S1 **Characterization of Tr17 cells.** Purified Foxp3.GFP^+^CD4^+^ T cells were cultured with anti-CD3/CD28 mAbs in the presence of IL-1β and IL-2 (**A**–**D**) or in absence of IL-1β and IL-2 (**A**) for 4 days. Expression of IL-17 (**A**), RORγt (**B**), Foxp3 (**C**) and Runx1 was examined. The results show mean value and SD of 3–5 independent experiments. (*) indicates p<0.05, and (**) indicates p<0.001.
(PDF)

Figure S2 **Helios is expressed in Tr17 cells.** (**A**) Expression of Helios in Freshly purified Foxp3.GFP^+^ cells was analyzed were stained with anti-Helios and Foxp3 antibodies followed by flow cytometry. (**B**) Foxp3.GFP^+^ cells were stimulated with anti-CD3/CD28 in the presence or absence of IL-1β and IL-2. Four days after the culture, the cells were stained with antibodies against Helios and IL-17.
(PDF)

Figure S3 **Downregulation of Runx1 expression upon activation of Treg cells.** Treg cells freshly isolated from Foxp3.GFP knock-in mice, Treg cells cultured in vitro with anti-CD3/CD28 mAbs and Treg cells cultured in vitro with anti-CD3/CD28 in the presence of IL-1β and IL-2 for 4 days were examined for expression of Runx1 by intracellular staining.
(PDF)

Figure S4 **RORγt and Runx1 but not Foxp3 bind to the** ***Il17*** **promoter and enhancer during Th17 differentiation.** Binding of endogenous RORγt, Runx1 and Foxp3 to the *Il17* promoter and enhancer was assessed by ChIP with antibodies against RORγt, Runx1 and Foxp3 in CD4^+^ T cells cultured for 4 d with aCD3/CD28 in the presence of TGF-β and IL-6 (Th17-polarizing conditions) and restimulated for 4 h with PMA and ionomycin. Results were normalized to input and are shown as -fold increase in signal relative to isotype control IgG used as ChIP negative control. Bar graphs show mean values and SD of triplicate samples from one out of two experiments.
(PDF)
