# Supplementary figures and images for: Runx1 and Runx3 Are Involved in the Generation and Function of Highly Suppressive IL-17-Producing T Regulatory Cells
Source: PLoS One. 2012 Sep 12;7(9):e45115. doi: 10.1371/journal.pone.0045115 (PMC3440330; doi:10.1371/journal.pone.0045115)

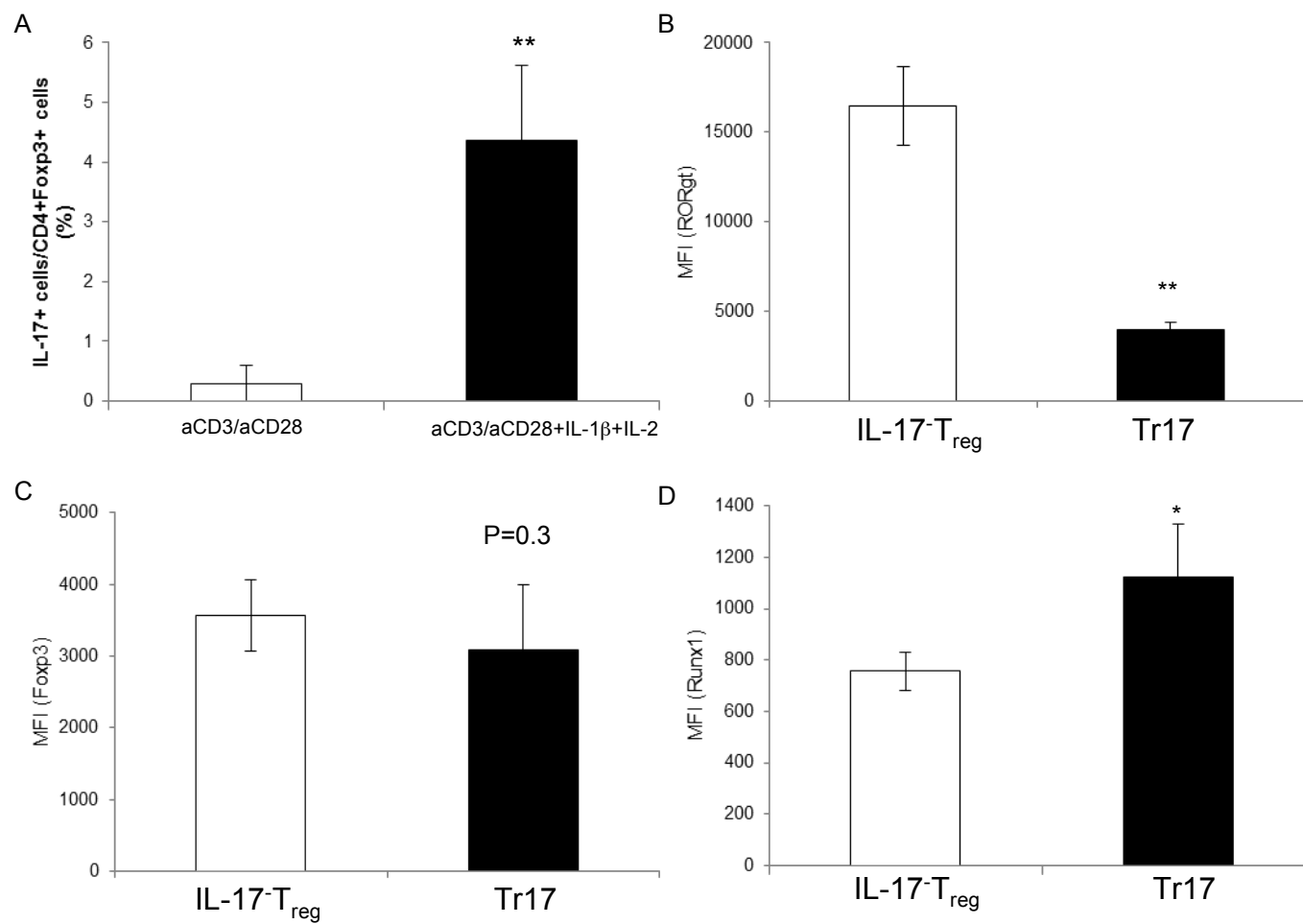

Figure S1

Supplement: Figure S1 — Characterization of Tr17 cells. Purified Foxp3.GFP+CD4+ T cells were cultured with anti-CD3/CD28 mAbs in the presence of IL-1β and IL-2 (A–D) or in absence of IL-1β and IL-2 (A) for 4 days. Expression of IL-17 (A), RORγt (B), Foxp3 (C) and Runx1 was examined. The results show mean value and SD of 3–5 independent experiments. (*) indicates p<0.05, and (**) indicates p<0.001. (PDF) [file pone.0045115.s001.pdf]

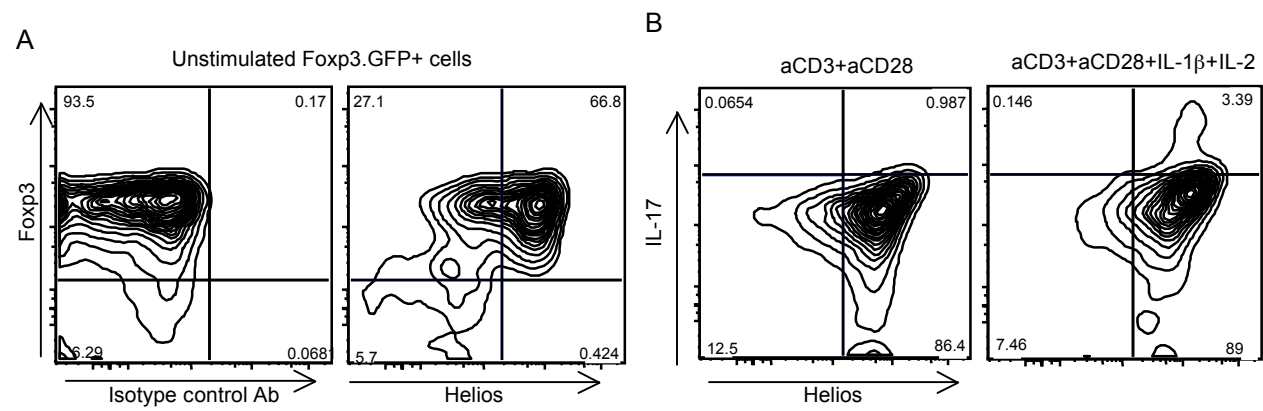

Figure S2

Supplement: Figure S2 — Helios is expressed in Tr17 cells. (A) Expression of Helios in Freshly purified Foxp3.GFP+ cells was analyzed were stained with anti-Helios and Foxp3 antibodies followed by flow cytometry. (B) Foxp3.GFP+ cells were stimulated with anti-CD3/CD28 in the presence or absence of IL-1β and IL-2. Four days after the culture, the cells were stained with antibodies against Helios and IL-17. (PDF) [file pone.0045115.s002.pdf]

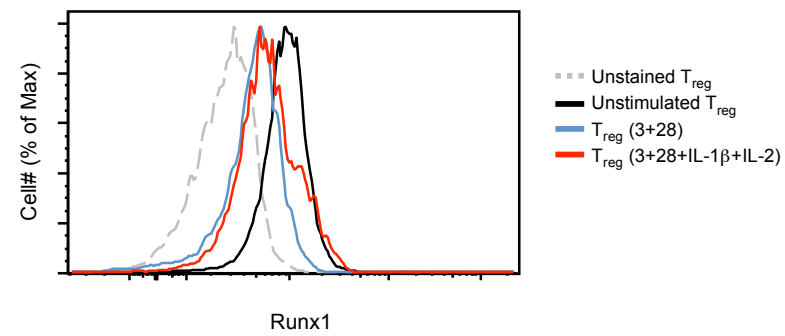

Figure S3

Supplement: Figure S3 — Downregulation of Runx1 expression upon activation of Treg cells. Treg cells freshly isolated from Foxp3.GFP knock-in mice, Treg cells cultured in vitro with anti-CD3/CD28 mAbs and Treg cells cultured in vitro with anti-CD3/CD28 in the presence of IL-1β and IL-2 for 4 days were examined for expression of Runx1 by intracellular staining. (PDF) [file pone.0045115.s003.pdf]

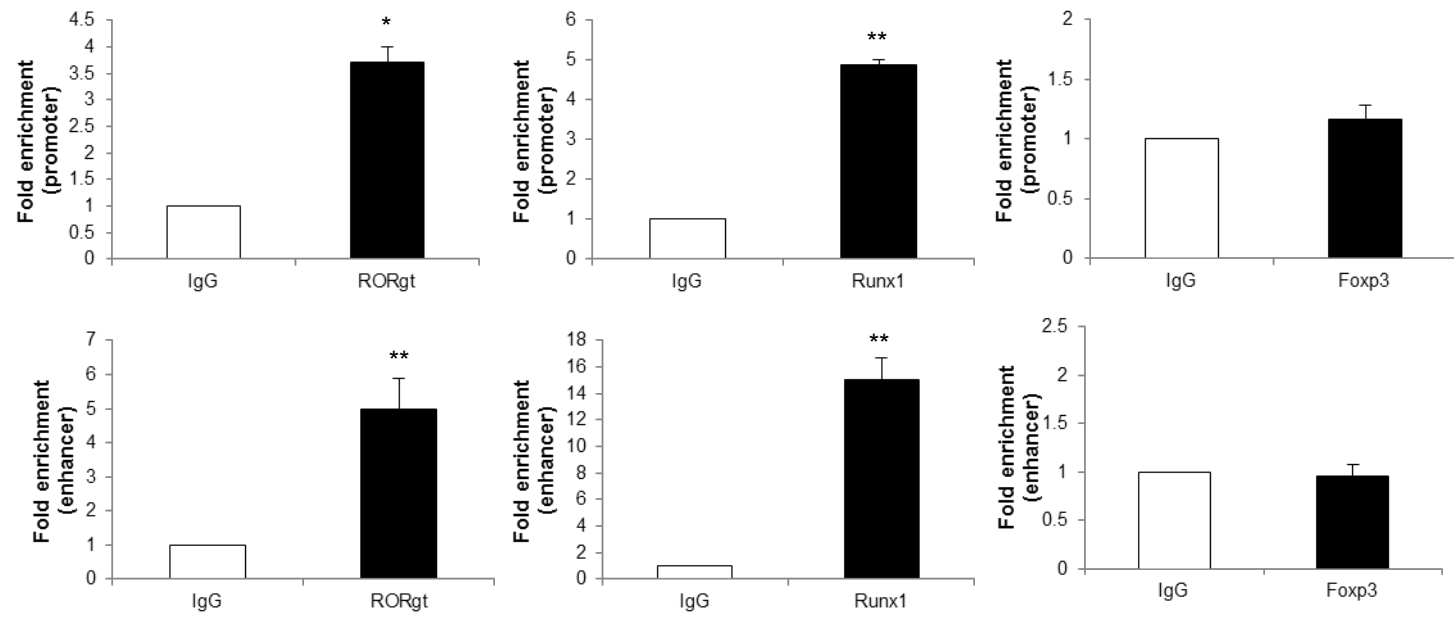

Figure S4

Supplement: Figure S4 — RORγt and Runx1 but not Foxp3 bind to the Il17 promoter and enhancer during Th17 differentiation. Binding of endogenous RORγt, Runx1 and Foxp3 to the Il17 promoter and enhancer was assessed by ChIP with antibodies against RORγt, Runx1 and Foxp3 in CD4+ T cells cultured for 4 d with aCD3/CD28 in the presence of TGF-β and IL-6 (Th17-polarizing conditions) and restimulated for 4 h with PMA and ionomycin. Results were normalized to input and are shown as -fold increase in signal relative to isotype control IgG used as ChIP negative control. Bar graphs show mean values and SD of triplicate samples from one out of two experiments. (PDF) [file pone.0045115.s004.pdf]
